# A giant apocrine hidrocystoma associated with elevated serum carcinoembryonic antigen levels: a case report

**DOI:** 10.1186/s13256-019-2175-8

**Published:** 2019-08-01

**Authors:** Kazuhiro Matsueda, Toshio Otani, Yusuke Fujioka, Motowo Mizuno

**Affiliations:** 1 Department of Gastroenterology and Hepatology, Kurashiki Central Hospital, 1-1-1 Miwa, Kurashiki, 710-8602 Japan; 2 Department of Dermatology, Kurashiki Central Hospital, 1-1-1 Miwa, Kurashiki, 710-8602 Japan; 3 Department of Plastic and Reconstructive Surgery, Kurashiki Central Hospital, 1-1-1 Miwa, Kurashiki, 710-8602 Japan

**Keywords:** High serum CEA level, Apocrine hidrocystoma

## Abstract

**Background:**

Serum carcinoembryonic antigen levels are often elevated in patients with malignant diseases. However, the etiology of elevated serum carcinoembryonic antigen levels may be extremely difficult to determine considering that this finding may occasionally occur in patients with benign diseases. Apocrine hidrocystomas, which are typically small and found on the face, are benign cystic lesions of apocrine sweat glands.

**Case presentation:**

A 58-year-old Japanese man was referred to us because of high serum carcinoembryonic antigen levels (15.9 ng/mL) found incidentally during a routine medical checkup. A physical examination revealed a hemispherical mass approximately 5 cm in diameter on his left thigh. Magnetic resonance imaging of the region showed a multilocular cystic mass with clear margins and a smooth surface, suggesting a cystic tumor. He underwent local mass resection. Pathological examination of the resected mass revealed an apocrine hidrocystoma with luminal cells, which tested immunohistochemically positive for carcinoembryonic antigen. Postoperatively, serum carcinoembryonic antigen levels returned to normal. This report is the first to describe an apocrine hidrocystoma associated with high serum carcinoembryonic antigen levels.

**Conclusions:**

An apocrine hidrocystoma can cause elevation of serum carcinoembryonic antigen levels. Despite its rarity, apocrine hidrocystoma should be considered in the differential diagnosis of conditions causing high serum carcinoembryonic antigen levels. In addition, skin diseases deserve more careful attention for patients with high serum carcinoembryonic antigen levels.

## Background

High serum carcinoembryonic antigen (CEA) levels may allow for an earlier diagnosis of gastrointestinal tract or lung malignancies because CEA-producing cells are said to be common among well-differentiated adenocarcinomas [1]. However, elevated CEA levels may occasionally occur in heavy tobacco smokers or in patients with benign diseases, such as diabetes mellitus, pulmonary tuberculosis [2], liver dysfunction, inflammatory bowel diseases [3], renal failure, and hypereosinophilic syndrome [4]. Therefore, it may be very difficult to determine the etiology of an elevated serum CEA level.

Apocrine hidrocystomas, which are typically small and found on the face during middle age or old age, are benign cystic lesions of apocrine sweat glands, [5–8].

In this case report, we present a case of an apocrine hidrocystoma in the thigh associated with high serum CEA levels. This report is the first to describe an apocrine hidrocystoma of the skin associated with high serum CEA levels.

## Case presentation

A 58-year-old Japanese man was referred to our hospital because of high serum CEA levels, found incidentally during a routine medical checkup. He was asymptomatic over the past year. His past medical history was significant for acute hepatitis A 20 years prior, which had been successfully treated. He is non-diabetic. He smoked 15 cigarettes per day until 10 years ago and does not consume alcohol.

A physical examination was unremarkable apart from a soft, non-tender, hemispherical mass approximately 5 cm in diameter on his left thigh. The mass had been present for approximately 40 years and had been gradually enlarging. Laboratory tests showed elevated serum CEA (15.9 ng/mL, normal < 5 ng/mL), but serum levels of carbohydrate antigen 19-9 (CA19-9) and calcitonin were not elevated. Liver function tests, thyroid function tests, and blood cell counts were normal. His serum glycated hemoglobin (HbA1c) and glucose levels were normal.

**Fig. 1:**
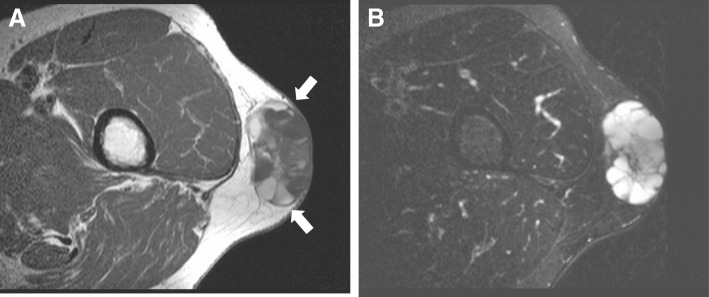
Magnetic resonance imaging showed a well-circumscribed multilocular cystic mass approximately 5 cm in diameter. The mass (*arrows*) comprised T1 low-signal (**a**) and T2 high-signal (**b**) intensity compartments

An upper gastrointestinal endoscopic examination, a chest and abdominal computed tomographic (CT) scan, and thyroid ultrasound demonstrated no abnormal findings. A colonic endoscopy showed two polyps, each 10 mm in size at the ascending colon and transverse colon, which were resected endoscopically. Histological examinations showed a focal high-grade tubular adenoma and a low-grade tubular adenoma, respectively. However, his serum CEA level increased to 30.4 ng/mL following the endoscopic resection. A fluorodeoxyglucose (FDG)-positron emission tomography (PET) scan showed no responsible masses. However, magnetic resonance imaging (MRI) of the region in his left thigh under the skin showed a well-circumscribed multilocular cystic mass with clear margins and a smooth surface. The mass comprised T1 low-signal (Fig. 1a) and T2 high-signal (Fig. 1b) intensity compartments with mostly fluid-fluid level. On the basis of these radiological findings, we suspected the mass to be a cystic tumor.

Our patient underwent local resection of the mass. Postoperatively, his serum CEA levels returned to normal. Ten years following surgery, his CEA levels remained normal without recurrence.

### Pathological findings

**Fig. 2:**
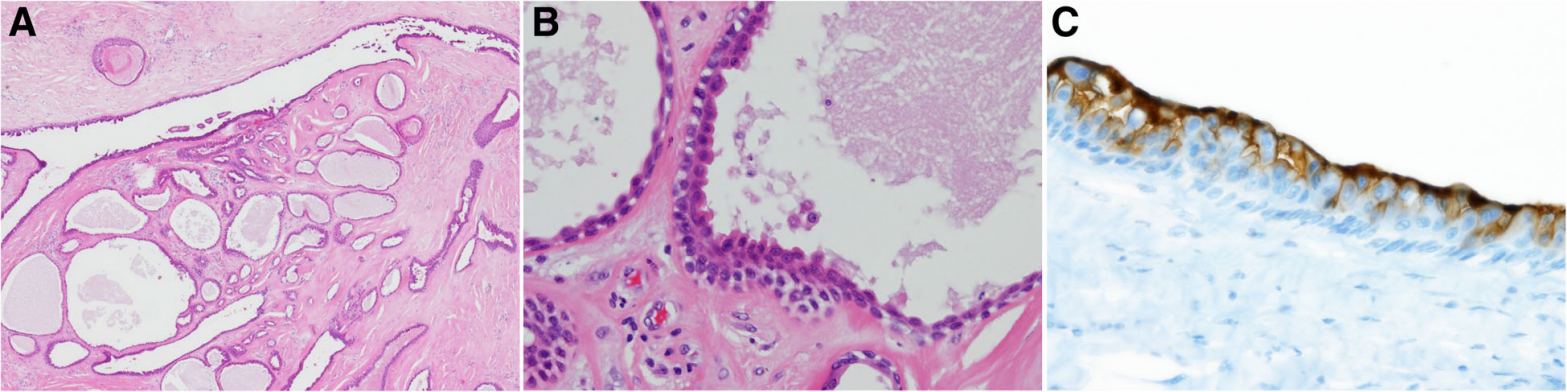
Histological findings of the operative specimens of the mass in the left thigh. **a** Multilocular cysts were revealed; hematoxylin and eosin staining, magnification × 40. **b** Multilocular cysts were lined by an almost double layer of cuboidal epithelium. Cells of the inner layer had abundant eosinophilic cytoplasm and apocrine snouts on the surface; hematoxylin and eosin staining, magnification × 200. **c** Carcinoembryonic antigen immunostaining was positive in the cytoplasm of luminal cells; magnification × 400

On gross examination, the mass was brownish, soft, and elastic, with a well-defined, dome-shaped, smooth surface. The mass measured 5.5 cm in diameter. Microscopic examination revealed multilocular cysts lined by an almost double layer of cuboidal epithelium (Fig. 2a). Cells of the inner layer had abundant eosinophilic cytoplasm and apocrine snouts on the surface (Fig. 2b). Squamous epithelium partially lined a portion of the cyst. CEA immunostaining was positive in the cytoplasm of luminal cells (Fig. 2c). These results confirmed a diagnosis of apocrine hidrocystoma.

## Discussion

Generally, there is no serum CEA threshold to differentiate benign and malignant conditions. CEA, which was originally speculated to occur exclusively in the fetal and neoplastic epithelial cells of the large bowel [9], is a glycoprotein found in several organs. In the skin, CEA is present exclusively in the sweat gland [6, 10–12]. Postoperatively, we confirmed that the high CEA level was due to apocrine hidrocystoma.

Apocrine hidrocystomas are histologically speculated to be benign cystic proliferations of apocrine glands. They are characterized by a double epithelial lining with decapitation secretion and an outer myoepithelial layer [6–8]. In the present case, immunohistochemical analysis revealed that the luminal cells of the apocrine hidrocystomas were CEA positive. Kariniemi *et al.* [13] reported that staining for CEA was found in 32 (64%), including two hidrocystomas, of 50 benign sweat gland tumors and CEA was occasionally found also in proliferating cells. Furthermore, Tokura *et al*. [14] demonstrated that all five cases of apocrine hidrocystomas examined were consistently positive for CEA in the luminal cells. In addition, no variations in staining pattern were observed among those cases. Although the reason for CEA production by these cells is unknown, it is possible that CEA may play a role in the innate immune defense and bind and trap microorganisms at the cell surface [15]. Therefore, CEA in the apocrine hidrocystoma may likewise play a similar role.

Apocrine hidrocystomas were first described by Mehregan in 1964 [7]. They appear as well-defined, dome-shaped, clear, and cystic nodules with smooth surfaces. Their color varies from flesh color to blue-black. They are generally located on the face, most often on the eyelid. However, rarely they can be found on the trunk or on the limbs, as in the present case [5, 16–18]. Their diameter ranges between 1 and 15 mm, and the giant type is a rare finding in the general population [5, 6]. A literature review identified exclusively eight cases of “a giant apocrine hidrocystoma,” including the present case [17–23]. Of the eight reported cases, seven were males and one was a female, with ages at presentation ranging between 29 and 70 years (mean, 52 years). The lesions were located as follows: four occurred on the face [19–22], two on the trunk [17, 18], one on the head [23], and one on the limb in the present case. Sizes ranged between 1.8 and 7 cm in diameter. In addition, three lesions, including the present case, exceeded 5 cm in diameter [17, 18]. Furthermore, in the seven cases, excluding the present one, serum CEA levels were not measured and tissue staining for CEA was not described. The present case is the first report of an apocrine hidrocystoma associated with high serum CEA levels. It is speculated that a large amount of CEA would be produced in cases of a giant apocrine hidrocystoma. Honma *et al.* reported a case of acquired idiopathic generalized anhidrosis with an elevated serum CEA, in which immunohistochemical analysis revealed CEA expression in eccrine sweat glands, and suggested that the increased serum CEA can be derived from sweat glands [24]. However, the correlation between lesion size and serum CEA level is unclear. This is due to the fact that previous reports have not examined serum CEA levels in patients with an apocrine hidrocystoma. We believe that the number of increasing cases may ascertain the correlation.

## Conclusions

In conclusion, an apocrine hidrocystoma can cause elevation of serum CEA levels. Despite its rarity, apocrine hidrocystomas should be considered in the differential diagnosis of conditions causing high serum CEA levels. In addition, skin diseases deserve more careful attention for patients with high serum CEA levels.

## Data Availability

All the data supporting our findings are contained within this report.

## Abbreviations

CA19-9: Carbohydrate antigen 19-9
CEA: Carcinoembryonic antigen
CT: Computed tomographic
FDG: Fluorodeoxyglucose
HbA1c: Glycated hemoglobin
MRI: Magnetic resonance imaging
PET: Positron emission tomography
